# Triglyceride–glucose index is associated with the risk of myocardial infarction: an 11-year prospective study in the Kailuan cohort

**DOI:** 10.1186/s12933-020-01210-5

**Published:** 2021-01-12

**Authors:** Xue Tian, Yingting Zuo, Shuohua Chen, Qian Liu, Boni Tao, Shouling Wu, Anxin Wang

**Affiliations:** 1 China National Clinical Research Center for Neurological Diseases, Fengtai District, Beijing Tiantan Hospital, Capital Medical University, No.119 South 4th Ring West Road, Beijing, 100070 China; 2 Department of Neurology, Beijing Tiantan Hospital, Capital Medical University, Beijing, China; 3 Department of Epidemiology and Health Statistics, School of Public Health, Capital Medical University, Beijing, China; 4 Beijing Municipal Key Laboratory of Clinical Epidemiology, Beijing, China; 5 Department of Cardiology, Kailuan Hospital, North China University of Science and Technology, 57 Xinhua East Rd, Tangshan, 063000 China; 6 Graduate School, North China University of Science and Technology, Tangshan, China; 7 School of Public Health, North China University of Science and Technology, Tangshan, China

**Keywords:** Triglyceride glucose index, Long-term exposure, Updated mean, Myocardial infarction, Risk factor

## Abstract

**Background:**

The triglyceride–glucose (TyG) index, which is a simple surrogate marker of insulin resistance, has been suggested as a contributor of cardiovascular disease. However, evidence on the effect of long-term elevation of the TyG index exposure on myocardial infarction (MI) is limited. The current study aimed to evaluate the association of baseline and long-term elevation of the TyG index exposure with the risk of MI.

**Methods:**

A total of 98,849 participants without MI at baseline (2006) were enrolled from the Kailuan study. The baseline TyG index was calculated as ln [fasting triglyceride (mg/dL) × fasting glucose (mg/dL)/2]. The long-term TyG index was characterized in two ways as follows. The updated mean TyG index was calculated as the mean of TyG index at all previous visits before MI occurred or the end of follow-up; alternatively, the TyG index was calculated as the number of visits with a high TyG index in 2006, 2008, and 2010, ranging from 0 (no exposure) to 3 (had high TyG index at all three study visits). Hazard ratio (HR) and 95% confidence interval (CI) was estimated using multivariable Cox proportion hazard models.

**Results:**

During a median follow-up of 11.03 years, 1555 incident MI occurred. In the multivariable-adjusted model, the risk of MI increased with quartiles of the baseline and updated mean TyG index, the HR in quartile 4 versus quartile 1 was 2.08 (95% CI,1.77–2.45) and 1.58 (1.18–2.12), respectively. Individuals with a high TyG index at all three visits had a 2.04-fold higher risk (95% CI, 1.63–2.56) of MI compared with no exposure. Subgroup analyses showed that the associations were more pronounced in women than in men (*P*_interaction_ = 0.0411).

**Conclusions:**

Elevated levels of the baseline and long-term TyG index are associated with an increased risk of MI. This finding indicates that the TyG index might be useful in identifying people at high risk of developing MI.

## Background

Myocardial infarction (MI) is the leading cause of morbidity and mortality of cardiovascular disease (CVD) worldwide, and it accounts for approximately one million deaths in China annually [1, 2]. Therefore, prevention of MI through understanding and reduction of risk factors has significant implications for public health and clinical practice.

Insulin resistance (IR) is a critical mechanism of the pathogenesis of diabetes mellitus, and has been extensively demonstrated to be a potential risk factor for CVD because it leads to atherosclerotic aneurysm and small vessel disease. Additionally, IR coexists with hypertension, obesity, and dyslipidemia, and all of these are well-known risk factors of CVD [3–9]. The triglyceride–glucose (TyG) index, which is derived from fasting triglyceride (TG) and fasting blood glucose (FBG) levels, has been proposed as a reliable surrogate marker of IR. Numerous studies have found a positive correlation between the TyG index and cardiovascular risk, including systematic arterial stiffness, carotid atherosclerosis, coronary artery calcification, coronary artery stenosis, symptomatic coronary artery disease, hypertension, and metabolic syndrome [10–16]. Furthermore, growing evidence has indicated that the TyG index is related to morbidity and mortality of CVD in the general population and many patient cohorts, including patients with and those without diabetes [14, 17–22].

Notably, however, the TyG index in most previous studies was measured only using a single time point and the long-term effect of a high TyG index remains unknown. To the best of our knowledge, the TyG index is affected by many biological and environmental factors, a single measurement of a high TyG index does not indicate that the body state has experienced a high TyG index for a long time. Therefore, a single measurement of this index may lead to incorrect classification of risk assessment for MI. Measurements of the TyG index at multiple time points, such as an updated mean or the number of visits with a high TyG index [23, 24], can characterize the longitudinal pattern of TyG index. These multiple measurements can provide a more robust assessment of associations with outcomes than analyses examining relationships using a single measurement of the TyG index, which may be attenuated with extended follow-up.

In the present study, we therefore aimed to investigate the association of an elevated TyG index at baseline and in the long term, where the long term TyG index was estimated as updated mean of TyG index and number of visits with high TyG index, with the risk of MI based on a large community-based prospective cohort study.

## Methods

### Study population

Data were deprived from the Kailuan study, which was a prospective cohort study that was conducted in the Kailuan community in Tanshan City, China. Details of the study design have been described previously [25–27]. In brief, a total of 101,510 participants (81,110 men and 20,400 women) aged 18–98 yeas agreed to participate and completed the first survey from June 2006 to October 2007 and underwent questionnaire assessments, physical examinations, and laboratory tests in the 11 hospitals responsible for health care of the Kailuan community. All participants were then followed up biennially to update the above-mentioned information. In the current study, we used the TyG index at baseline (2006) to predict the subsequent risk of MI. Data analysis was performed from 1 January 2006 to 31 December 2017. We excluded 1317 participants with previous MI and 1344 participants with missing data of TG and FBG levels at baseline. A total of 98,849 participants were enrolled for the analysis of the baseline and updated mean TyG index. In analysis of the number of visits with high a TyG index, we further excluded 42,694 participants without completed data on TG and FBG levels and 703 participants who had MI during 2006 and 2010. Therefore, 55,452 participants were included for the current analysis (Additional file 1: Figure S1).

### Data collection and definitions

Information on demographics, socioeconomic status, medical history, and lifestyle was collected using a self-report questionnaire by trained staffs, including age, sex, education, income, smoking status, drinking status, physical activity, and a history of disease and medication. Education was classified as illiteracy or primary school, middle school, and high school or above. Income was categorized into > 800 and ≤ 800 yuan/month. Smoking and drinking habits were stratified into never, former or current. Physical activity was classified as ≥ 4 times per week and ≥ 20 min at a time, < 80 min per week, or none. Blood pressure was measured in the in the seated position using a mercury sphygmomanometer, and the average of three readings was calculated as systolic blood pressure (SBP) and diastolic blood pressure (DBP).

Fasting blood samples were collected from the antecubital vein after an 8- to 12-h overnight fast. All the plasma samples were assessed using an auto-analyzer (Hitachi 747, Tokyo, Japan) at the central laboratory of Kailuan Hospital. FBG levels were measured using the hexokinase/glucose-6-phosphate dehydrogenase method with the coefficient of variation using blind quality control specimens < 2.0%. Serum total cholesterol (TC), TG, low-density lipoprotein cholesterol (LDL-C), and high-density lipoprotein cholesterol (HDL-C) levels were measured with the enzymatic colorimetric method. High-sensitivity C-reactive protein (hs-CRP) levels were measured with high-sensitivity particle-enhanced immunonephelometry assay. Diabetes was defined as FBG levels ≥ 7.0 mmol/L, any use of glucose-lowing drugs, or any self-reported history of diabetes. Hypertension was defined as SBP ≥ 140 mm Hg or DBP ≥ 90 mm Hg, any use of antihypertensive drugs, or a self-reported history of hypertension. Dyslipidemia was defined as any self-reported history or use of lipid-lowering drugs, or TC levels ≥ 5.17 mmol/L or TG levels ≥ 1.69 mmol/L or LDL-C levels ≥ 3.62 mmol/L or HDL-C level ≤ 1.04 mmol/L.

### Calculation of the baseline and long-term TyG index

The TyG index was calculated as ln [(fasting TG (mg/dl) × FBG (mg/dl)/2] [21]. To determine long-term TyG index patterns of individuals, we calculated the updated mean TyG index and the number of visits with a high TyG index. The updated mean TyG index was calculated using all available TyG index measurements from baseline to the year before MI occurred or to the end of follow-up (2017). An example of this calculation is that incident MI in 2013 was related to the average concentration of TyG index level in 2006, 2008, 2010, and 2012. Those participants who did not develop MI before the end of follow-up (2017) had six times of TyG index measurements and those who developed MI had ≤ six TyG index measurements.

In the current study, a high TyG index was defined as a TyG index greater the cutoff value, which was estimated by the receiver operating characteristic curve (ROC). Then the number of visits with a high TyG index was calculated using the TyG index value at visits in 2006, 2008 and 2010. We assigned 1 point to a TyG index higher than the cutoff value, and 0 point for TyG index less than the cutoff value at each visit. Therefore, the number of visits with high a TyG index was ranged from 0 to 3. An example of this calculation is that 2 points represent a high TyG index twice in the 3 visits.

### Follow-up and assessment of MI

Participants were followed via face-to-face interviews at every 2-year routine medical examination until 31 December 2017 or death. The primary outcome was the first occurrence of MI, either the first nonfatal MI event or death due to MI without a history of MI. The diagnosis of MI events was obtained from biennial personal interviews, the discharge summary from the 11 hospitals, and medical records from medical insurance, using the International Classification of Disease, 10th Revision code I21 for MI. Diagnosis of MI was based on combinations of chest pain symptoms, electrocardiographic signs, and cardiac enzyme levels. The criteria were consistently applied across all 11 hospitals [28].

### Statistical analysis

Continuous variables are described as median and interquartile range (IQR) owing the skewed distribution. Categorical variables are described as frequencies and percentages. The Wilcoxon or Kruskal–Wallis test was used to analyze group differences for continuous variables, and the chi-square test was used for categorical variables. Person-years were determined from the date when the message was collected at baseline to either the date of onset of MI, death, or the date of participating in the last examination in this analysis, whichever came first. The Kaplan–Meier method was performed to evaluate the incidence rate of MI and differences among groups were evaluated by the log-rank test.

Three multivariate Cox proportional hazard regressions were constructed to estimate the association of the baseline and long-term TyG index with the risk of MI by calculating the hazard ratio (HR) and 95% confidence interval (CI). The validity of the proportionality assumption was verified by scaled Schoenfeld residuals for the baseline TyG index and the number of visits with a high TyG index, and by including a time-dependent covariate with an interaction of the TyG index and a logarithmic function of survival time in the model for the updated mean TyG index. The results of which suggested that the assumptions were not violated (Additional file 1: Table S1). Model 1 was adjusted for age and sex at baseline; Model 2 was further adjusted for education, income, smoking, alcohol abuse, physical activity, and BMI at baseline; Model 3 was further adjusted for SBP, DBP, a history of hypertension, diabetes mellitus, and dyslipidemia, antidiabetic drugs, lipid-lowering drugs, antihypertensive drugs, and HDL-C, LDL-C, and hs-CRP levels at baseline. We added an interaction between the updated mean TyG index and a logarithmic function of survival time in the above-mentioned model for the analysis of the updated mean TyG index and MI. The *P* values for trend were computed using quartiles or the number of visits with a high TyG index as ordinal variables. We also analyzed the effect of baseline and the updated mean TyG index on MI as a continuous variable using a restricted cubic spline with 5 knots (at the 5th, 25th, 50th, 75th, and 95th percentiles). The optimum cutoff value of the TyG index in case of incident MI was determined using the ROC curve analysis. The best cutoff point for the TyG index was assessed by the maximum value of the Youden index, which was calculated as sensitivity + specificity − 1.

Additional analyses were performed to evaluate the robustness of the association of the TyG index with the risk of MI. First, stratified analyses according to baseline age (< 60 and ≥ 60 years), sex, diabetes (no and yes), and BMI (< 28 and ≥ 28 kg/m^2^) were used to examine the consistence of the effect of a high TyG index (quartile 4 group) for the risk of MI. Second, to evaluate the robustness of our main results, sensitivity analysis was conducted by excluding all deaths during the follow-up period (n = 9640). Another sensitivity analysis was performed by excluding participants with abnormal FBG levels (≥ 7.0 mmol/L) or abnormal TG levels (≥ 1.7 mmol/L) at baseline (n = 35 346).

All analyses were conducted using SAS version 9.4 (SAS Institute Inc., Cary, NC, USA). A two-sided *P* < 0.05 was considered statistically significant.

## Results

### Baseline characteristics

A total of 98 849 participants were included in the current study. The median age was 51.81 years (IQR, 43.60–59.23) and 78 825 (79.75%) were men. Baseline characteristics by quartiles of the TyG index are shown in Table 1. Participants with a higher TyG index tended to be older, men, less educated, have higher income, more current smokers and alcohol drinkers, a higher prevalence of diabetes, hypertension, and dyslipidemia, take more antihypertensive, antidiabetic, and lipid-lowering drugs, have higher SBP, DBP, FBG, TC, TG, LDL-C, HDL-C, and hs-CRP levels compared with participants in quartile 1 group. Similar results were observed when participants were categorized by the updated mean TyG index (Additional file 1: Table S2).

**Table 1:** Baseline characteristics of participants according to quartiles of TyG index

Characteristics	Overall	Quartiles of TyG index				*P* value
		Q1	Q2	Q3	Q4	
No. of participants	98,849	24,712	24,716	24,708	24,713	
TyG index	8.58 (8.18–9.06)	7.91 (7.70–8.06)	8.39 (8.29–8.48)	8.79 (8.68–8.91)	9.46 (9.23–9.82)	< 0.0001
Age, years	51.81 (43.60–59.23)	50.77 (41.81–58.91)	51.82 (43.60–59.18)	52.36 (44.22–59.70)	52.16 (44.44–58.96)	< 0.0001
Men, n (%)	78,835 (79.75)	18,283 (73.98)	19,634 (79.44)	20,104 (81.37)	20,814 (84.22)	< 0.0001
High school or above, n (%)	19,189 (20.09)	5781 (24.32)	4586 (19.12)	4561 (19.12)	4261 (17.84)	< 0.0001
Income > 800 RMB/month, n (%)	13,659 (14.31)	3699 (15.57)	3223 (13.45)	3357 (14.08)	3380 (14.16)	< 0.0001
Body mass index, kg/m^2^	24.84 (22.60–27.22)	23.14 (21.11–25.34)	24.44(22.39–26.64)	25.43 (23.4–27.67)	26.30 (24.22–28.41)	< 0.0001
Systolic blood pressure, mm Hg	130.00 (119.30–141.30)	120.00 (110.00–136.00)	129.30 (117.30–140.00)	130.00 (120.00–145.00)	132.00 (120.00–150.00)	< 0.0001
Diastolic blood pressure, mm Hg	80.00 (78.70–90.00)	80.00 (70.70–85.00)	80.00 (77.30–90.00)	81.00 (79.30–90.00)	85.00 (80.00–92.00)	< 0.0001
Current smoker, n (%)	33,064 (34.34)	8014 (33.50)	7750 (32.10)	8284 (34.39)	9016 (37.37)	< 0.0001
Current alcohol use, n (%)	35,945 (37.32)	8941 (37.36)	8415 (34.85)	8921 (37.02)	9668 (40.07)	< 0.0001
Active physical activity, n (%)	87,005 (91.28)	21,660 (91.33)	21,964 (91.77)	21,641 (90.85)	21,740 (91.19)	0.0046
Diabetes Mellitus, n (%)	3033 (3.07)	182 (0.74)	348 (1.41)	667 (2.70)	1836 (7.43)	< 0.0001
Hypertension, n (%)	12,300 (12.44)	1899 (7.685)	2549 (10.31)	3479 (14.08)	4373 (17.70)	< 0.0001
Dyslipidemia, n (%)	5757 (5.82)	751 (3.04)	1044 (4.22)	1613 (6.53)	2349 (9.50)	< 0.0001
Antihypertensive drugs, n (%)	2311 (2.34)	137 (0.55)	246 (1.00)	513 (2.08)	1415 (5.73)	< 0.0001
Antidiabetic drugs, n (%)	845 (0.85)	97 (0.39)	156 (0.63)	201 (0.81)	391 (1.58)	< 0.0001
Lipid-lowering drugs, n (%)	10,670 (10.79)	1647 (6.66)	2188 (8.85)	3050 (12.34)	3785 (15.32)	< 0.0001
Fasting plasma glucose, mmol/L	5.11 (4.66–5.71)	4.78 (4.37–5.20)	5.01 (4.62–5.46)	5.24 (4.80–5.82)	5.67 (5.02–6.90)	< 0.0001
Total cholesterol, mmol/L	4.92 (4.28–5.59)	4.59 (4.03–5.20)	4.89 (4.29–5.46)	5.07 (4.47–5.71)	5.19 (4.44–5.94)	< 0.0001
Triglycerides, mmol/L	1.27 (0.89–1.93)	0.70 (0.58–0.82)	1.10 (0.98–1.22)	1.56 (1.36–1.79)	2.76 (2.18–3.90)	< 0.0001
HDL-C, mmol/L	1.50 (1.28–1.76)	1.54 (1.30–1.80)	1.52 (1.31–1.77)	1.48 (1.27–1.74)	1.47 (1.25–1.74)	< 0.0001
LDL-C, mmol/L	2.33 (1.82–2.83)	2.13 (1.60–2.72)	2.37 (1.91–2.82)	2.41 (1.97–2.90)	2.38 (1.82–2.88)	< 0.0001
Hs-CRP, mg/dL	0.80 (0.30–2.20)	0.60 (0.21–1.90)	0.73 (0.30–2.00)	0.90 (0.34–2.20)	1.04 (0.40–2.66)	< 0.0001

*LDL-C* low-density lipoprotein cholesterol, *HDL-C* high-density lipoprotein cholesterol, *hs-CRP* high-sensitivity C-reactive protein, *TyG* triglyceride glucose

### Association of the baseline and updated mean TyG index with the risk of MI

**Fig. 1:**
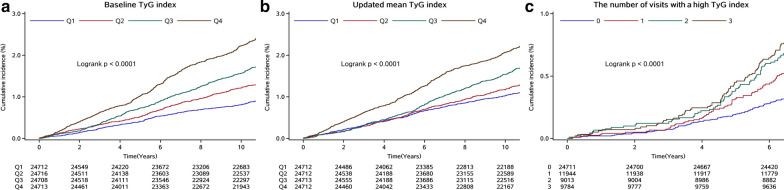
Kaplan–Meier estimation of myocardial infarction by baseline and updated mean of TyG index. *TyG* triglyceride-glucose

During a median follow-up of 11.03 years (IQR, 10.71–11.21), 1555 (1.57%) participants developed MI. The association between the TyG index and the risk of MI is shown in Table 2. The incidence of MI substantially increased with increasing TyG index quartiles, from 0.84 (95% CI, 0.74–0.96) in quartile 1 to 2.28 (95% CI, 2.11–2.48) per 1000 person-years in quartile 4. Kaplan–Meier curves also showed that participants in quartile 4 of the baseline and updated mean TyG index had a higher risk for MI events than participants in the other groups during the follow-up period (log-rank test, *P* < 0.0001; Fig. 1a and b).

**Table 2:** HR (95% CI) for risk of myocardial infarction according to quartiles of the TyG index

Variables	Quartiles of the TyG index				Per 1 unit increase	*P* for trend
	Q1	Q2	Q3	Q4		
*Baseline TyG index*						
Case, n (%)	220 (0.89)	315 (1.27)	430 (1.74)	590 (2.39)		
Incidence rate, per 1000 person-y	0.84 (0.74–0.96)	1.21 (1.08–1.35)	1.65 (1.51–1.82)	2.28 (2.11–2.48)		
Model 1	Reference	1.39 (1.17–1.65)	1.88 (1.60–2.21)	2.69 (2.30–3.14)	1.61 (1.51–1.72)	< 0.0001
Model 2	Reference	1.33 (1.12–1.58)	1.73 (1.47–2.05)	2.41 (2.06–2.83)	1.54 (1.43–1.65)	< 0.0001
Model 3	Reference	1.26 (1.06–1.50)	1.60 (1.36–1.89)	2.08 (1.77–2.45)	1.43 (1.33–1.53)	< 0.0001
Sensitivity analysis^a^	Reference	1.24 (1.01–1.51)	1.62 (1.34–1.96)	2.02 (1.67–2.44)	1.39 (1.28–1.52)	< 0.0001
Sensitivity analysis^b^	Reference	1.22 (1.02–1.46)	1.50 (1.24–1.82)	3.60 (1.48–8.80)	1.63 (1.32–2.02)	< 0.0001
*Updated mean TyG index*						
Case, n (%)	264 (1.07)	318 (1.29)	421 (1.70)	552 (2.23)		
Incidence rate, per 1000 person-y	1.02 (0.90–1.15)	1.22 (1.09–1.36)	1.61 (1.46–1.36)	2.13 (1.96–2.32)		
Model 1	Reference	1.20 (1.00–1.43)	1.54 (1.24–1.94)	2.10 (1.57–2.79)	1.74 (1.48–2.04)	< 0.0001
Model 2	Reference	1.13 (0.95–1.36)	1.42 (1.14–1.78)	1.86 (1.39–2.49)	1.64 (1.39–1.93)	< 0.0001
Model 3	Reference	1.07 (0.90–1.29)	1.30 (1.03–1.62)	1.58 (1.18–2.12)	1.49 (1.26–1.76)	< 0.0001
Sensitivity analysis^a^	Reference	1.01 (0.78–1.20)	1.24(1.02–1.58)	1.42 (1.13–2.05)	1.47 (1.32–1.63)	< 0.0001
Sensitivity analysis^b^	Reference	1.17 (0.86–1.59)	1.21 (0.90–1.64)	1.80 (1.36–2.38)	1.86 (1.25–2.57)	< 0.0001

*CI* confidence interval, *HR* hazard ratio, *TyG* triglyceride glucose

Model 1, adjusted for age and sex at baseline

Model 2, adjusted for variables in model 1 plus level of education, income, smoking, alcohol abuse, physical activity, and BMI at baseline

Model 3, adjusted for variables in model 2 plus SBP, DBP, a history of hypertension, diabetes mellitus, and dyslipidemia, antidiabetic drugs, lipid-lowering drugs, antihypertensive drugs, HDL-C, LDL-C, and hs-CRP at baseline

^a^Sensitivity analysis was adjusted for variables in model 3 and further excluded all deaths during the follow-up visits

^b^Sensitivity analysis was excluded those with abnormal FBG (≥ 7.0 mmol/L) or abnormal TG level (≥ 1.7 mmol/L) at baseline, and adjusted for covariates in Model 3

The risk of MI increased over time by baseline TyG quartiles and remained significant even after adjustment for potential confounding factors, the fully adjusted HRs (model 3) were 1.26 (95% CI, 1.06–1.50), 1.60 (1.36–1.89), and 2.08 (1.77–2.45) for quartiles 2, 3 and 4, respectively, versus quartile 1 of the baseline TyG index (*P* for trend < 0.0001). The association remained significant in analyses using the updated mean TyG index, the adjusted HR in quartile 4 versus quartile 1 was 1.58 (95% CI, 1.18–2.12, *P* for trend < 0.0001; Table 2). Sensitivity analysis yielded the similar results when all the deaths during the follow-up period were excluded, and when the analysis was restricted to participants with normal FBG and TG levels.

**Fig. 2:**
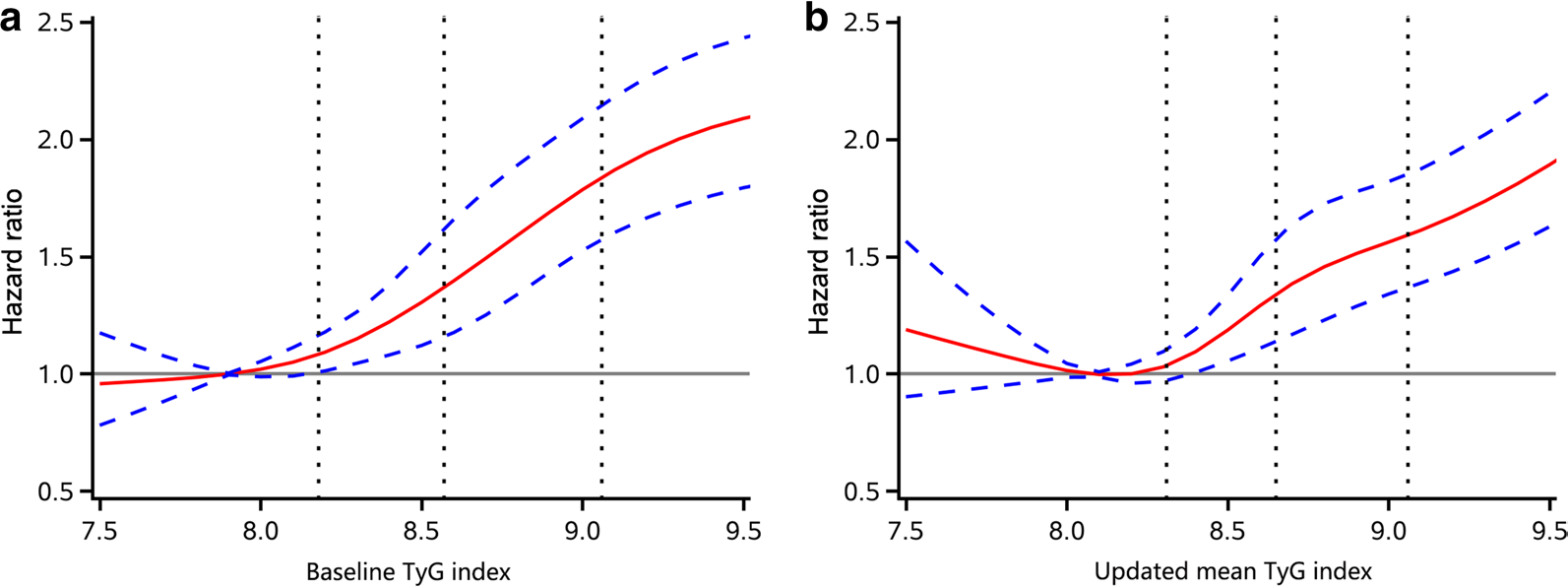
Multivariable-adjusted hazard ratios for MI based on restricted cubic spines with 5 knots at 5th, 25th, 50th, 75th, and 95th percentiles of **a** baseline TyG index and **b** updated mean TyG index. Abbreviation: TyG, triglyceride-glucose. Red line represent references for hazard ratios, and blue lines represent 95% confidence interval. Adjusted for age, sex, education, income, smoking, alcohol abuse, physical activity, and body mass index, systolic blood pressure, diastolic blood pressure, a history of hypertension, diabetes mellitus, and dyslipidemia, antidiabetic drugs, lipid-lowering drugs, antihypertensive drugs, HDL cholesterol, LDL cholesterol, and hs-CRP

When the TyG index was treated as a continuous variable, a per 1 unit increase of the baseline TyG index was associated with a 43% higher risk of MI (HR, 1.43; 95% CI, 1.33–1.53). Multivariable adjusted spline regression models showed a J-shaped association between the baseline TyG index and the risk of MI (Fig. 2a). All the significant results persisted in the analyses using the updated mean TyG index (Table 2, Fig. 2b).

### Association between the number of visits with a high TyG index and the risk of MI

In this analysis, we compared baseline characteristics of participants who were included and those who were excluded owing to missing data (Additional file 1: Table S3). There were significant clinical differences regarding baseline characteristics of age, sex, education, SBP, DBP, drinking status, and LDL-C levels between the two groups.

Among the 55,452 enrolled participants, the area under the curve of the TyG index for MI was 0.59 (95% CI 0.57–0.60). The cutoff value we determined for the TyG index for incident MI was 8.83 (Additional file 1: Figure S2). Participants with all three study visits with a high TyG index had a higher risk of MI than the other groups (log-rank test, *P* < 0.0001, Fig. 1c). Significant associations remained even after adjustment for potential confounders, the HRs in the fully adjusted model were 1.54 (95% CI, 1.23–1.93), 1.78 (1.41–2.26), and 2.04 (1.63–2.56) for participants with a high TyG index once, twice, three times of the three visits, respectively, compared to those with a TyG index less than the cutoff value at all the three visits. Sensitivity analysis yielded similar results (Table 3).

**Table 3:** HR (95% CI) for risk of myocardial infarction according to number of visits with a high TyG index

Variables	Number of visits with a high TyG index*				
	0	1	2	3	*P* for trend
Case, n (%)	173 (0.70)	139 (1.40)	126 (1.40)	164 (1.68)	
Incidence rate, per 1000 person-y	0.65 (0.56–0.75)	1.07 (0.91–1.27)	1.29 (1.09–1.54)	1.55 (1.33–1.81)	
Model 1	Reference	1.69 (1.36–2.12)	2.10 (1.67–2.64)	2.52 (2.03–3.12)	< 0.0001
Model 2	Reference	1.61 (1.28–2.01)	1.90 (1.50–2.40)	2.21 (1.77–2.76)	< 0.0001
Model 3	Reference	1.54 (1.23–1.93)	1.78 (1.41–2.26)	2.04 (1.63–2.56)	< 0.0001
Sensitivity analysis	Reference	1.48 (1.16–1.91)	1.78 (1.37–2.31)	2.16 (1.69–2.78)	< 0.0001

*CI* confidence interval, *HR* hazard ratio, *TyG* triglyceride glucose

^*^High TyG index was defined as TyG index ≥ 8.83

Model 1, adjusted for age and sex at baseline

Model 2, adjusted for variables in model 1 plus level of education, income, smoking, alcohol abuse, physical activity, and BMI at baseline

Model 3, adjusted for variables in model 2 plus SBP, DBP, a history of hypertension, diabetes mellitus, and dyslipidemia, antidiabetic drugs, lipid-lowering drugs, antihypertensive drugs, HDL-C, LDL-C, and hs-CRP at baseline

Sensitivity analysis was adjusted for variables in model 3 and further excluded all deaths during the follow-up visits

### Subgroup analysis

Results of subgroup analyses are shown in Table 4. Generally, a high TyG index (quartile 4) was significantly associated with the risk of MI across various subgroups. There was a significant interaction in the sex subgroup (*P* for interaction = 0.0411 in model 3). The hazards of a high TyG index on MI were more prominent in women (HR, 3.77; 95% CI, 1.86–7.64) than men (HR, 1.93; 95% CI, 1.63–2.29).

**Table 4:** Subgroup analysis for the risk of myocardial infarction by baseline TyG index (impact of TyG Q4)

Variables	Group	Model 1		Model 2		Model 3	
		HR (95% CI)	*P*_interaction_	HR (95% CI)	*P*_interaction_	HR (95% CI)	*P*_interaction_
*Age*							
< 60 years	Q4	2.69 (2.15–3.35)	0.1428	2.39 (1.90–3.00)	0.2935	2.12 (1.69–2.68)	0.3000
≥ 60 years	Q4	2.43 (1.95–3.02)		2.25 (1.79–2.83)		1.94 (1.53–2.45)	
*Sex*							
Women	Q4	5.29 (2.70–10.37)	0.0128	5.13 (2.59–10.18)	0.0164	3.77 (1.86–7.64)	0.0411
Men	Q4	2.47 (2.11–2.90)		2.20 (1.86–2.60)		1.93 (1.63–2.29)	
*Diabetes*							
No	Q4	2.57 (2.19–3.02)	0.1352	2.28 (1.93–2.69)	0.1430	2.09 (1.77–2.47)	0.1138
Yes	Q4	1.56 (0.68–3.57)		1.64 (0.71–3.80)		1.54 (0.66–3.57)	
*BMI*							
< 28 kg/m^2^	Q4	2.65 (2.23–3.14)	0.3394	2.43 (2.04–2.90)	0.2573	2.08 (1.74–2.49)	0.4420
≥ 28 kg/m^2^	Q4	2.30 (1.48–3.58)		2.30 (1.48–3.58)		2.07 (1.32–3.23)	

*BMI* body mass index, *CI* confidence interval, *HR* hazard ratio, *TyG* triglyceride glucose

Model 1, adjusted for age and sex at baseline other than the variable for stratification

Model 2, adjusted for variables in model 1 plus level of education, income, smoking, alcohol abuse, physical activity, and BMI at baseline other than the variable for stratification

Model 3, adjusted for variables in model 2 plus SBP, DBP, a history of hypertension, diabetes mellitus, and dyslipidemia, antidiabetic drugs, lipid-lowering drugs, antihypertensive drugs, HDL-C, LDL-C, and hs-CRP at baseline other than the variable for stratification

## Discussion

In this large community-based cohort study, we observed that a high baseline and long-term TyG index were associated with the risk of MI, even in those with normal FBG and TG levels. Specifically, participants in the quartile 4 group of the baseline and updated mean TyG index, and those had three times of a high TyG index had a higher risk of MI than the other groups. Additionally, subgroup analyses showed there was a significant interaction between sex and the TyG index, the effect of a high TyG index on the risk of MI was more pronounced in women compared with men. The results obtained from this large, prospective, observational study have important implications for prevention of MI.

IR is defined as a decrease in the efficiency of insulin in promoting glucose uptake and utilization and has been considered as an important risk factor for CVD [3–9]. Evaluations of IR requires sophisticated methods, which are not available for use in daily clinical practice [11]. Therefore, a number of surrogate markers of IR have been proposed and compared with the gold standard of the hyperinsulinemic–euglycemic clamp [29]. Hemeostasis model assessment of IR (HOMA-IR), which is calculated by fating insulin and glucose, is commonly used for testing IR. However, the insulin concentrations are not routinely measured in clinical practice, leading to an HOMA-IR that is inappropriate for clinical practice on a large scale. Therefore, researchers began to study the TyG index, which is a simple, cost-effective, reproducible, reliable, and valid surrogate marker of IR. Additionally, the TyG index is well related to the hyperinsulinemic–euglycemic clamp and HOMA-IR [30, 31].

Our finding that a high baseline TyG index was associated with an increased risk of developing MI is supported by several cross-sectional, case–control, prospective ad retrospective studies in general and patients cohorts [14, 17–22]. The National Health and Nutrition Examination Survey III study of 6093 participants showed that a high TyG index had a 21% higher risk of subclinical myocardial injury [20]. The Vascular Metabolic CUN cohort study of 5 014 patients suggested that the TyG index can be used to identify high-risk cardiovascular events at an early stage with a HR of 2.32 (95% CI: 1.65–3.26) for the highest quintile [17]. Another retrospective cohort study including 6078 subjects aged over 60 years demonstrated that an the risk of developing CVD was 1.72-fold higher in the quartile 4 group of TyG index, compared with quartile 1 [18]. Two Chinese cohort studies reported that the TyG index was associated with adverse cardiovascular outcomes in patients with type 2 diabetes mellitus or with acute coronary syndrome who underwent percutaneous coronary intervention [21, 22]. In line with previous studies, our study of 98,849 participants with sufficient statistical power showed that individuals in the quartile 4 group of the baseline TyG index had a 2.08-fold higher risk of MI compared with the quartile 1 group. After adjusted all potential covariates, this significant association of a higher risk of MI in the quartile 4 group persisted in those with normal FBG and TG levels.

Most previous studies relied on TyG index measurements from a single time point, which is an important limitation because of variability of the TyG index level over time. Therefore, measurements of long-term exposure of TyG provide more reliable and robust results. In the current study, the long-term TyG index was estimated at multiple time points with the updated mean TyG index and the number of visits with a high TyG index. Recent studies have used these methods of other parameters to predict CVD as follows. The Clinical Practice Research Datalink study showed that time-updated hemoglobin A_1c_ values showed a stronger relation with MI than baseline hemoglobin A_1c_ values [32]. Another Kailuan study revealed that participants with three visits with high hs-CRP levels had a 38% higher risk of CVD and 1.13 higher risk of MI than those without high hs-CRP levels [33]. Using the methods mentioned above, our study showed that the risk of MI increased with quartiles of the updated mean TyG index and the number of visits with a high TyG index. This finding indicated that a high TyG index in the long term was also associated with the risk of MI. Taken together, our findings suggested that not only a high baseline TyG index, but also a high TyG index in the long term, can predict a high risk of MI. Therefore, monitoring the TyG index over time in clinical practice is important.

Another important finding of our study is that the association between the TyG index and the risk of MI was more pronounced in women than men, which is in accordance with previous studies [34, 35]. A cohort study showed that higher fasting serum insulin levels and HOMA-IR were associated with incident hypertension in women, but not in men [34]. Moreover, the Coronary Artery Calcification in Type 1 Diabetes cohort study found that type 1 diabetes affected adipose and skeletal muscle insulin sensitivity to a greater extent in women than in men, which might have contributed to the greater relative increase in cardiovascular risk in women [35]. A possible mechanism for this finding may be a role of estrogen in premenopausal cardiovascular protection and enhanced insulin sensitivity. Following menopause, women’s cardiovascular protection decreases and IR increases [36].

Although the potential mechanism underlying the association of the TyG index with the risk of MI has not been elucidated, there are several speculations summarized as follows. First, studies have shown that FBG levels mainly reflects IR from the liver, whereas fasting TG levels mainly reflect IR from adipose cells. Therefore, the TyG index may reflect IR from two aspects. IR is significantly associated with endothelial dysfunction, oxidative stress, cardiovascular remodeling, coagulation imbalance, and the inflammation response [4, 20, 37]. Second, the TyG index is linked to IR, which can induce an imbalance in glucose metabolism that generates chronic hyperglycemia, and can also alter lipid metabolism and lead to dyslipidemia. These metabolic changes may contribute to the development of MI [4]. Third, the TyG index is related to arterial stiffness as evaluated by pulse pressure, brachial–ankle pulse wave velocity, and carotid–femoral pulse wave velocity, all of which are major risk factors of MI [10, 11].

## Strengths and limitations

The strengths of the study include its prospective design, large community-based sample, long follow-up period, and the availability of repeated measurements of the TyG index. However, the current study also has several limitations. First, owing to the observational nature of the study, we could not establish a causal association between the TyG index and the risk of MI. Therefore, our findings need to be confirmed in future studies. Furthermore, although potential cardiac risk factors were adjusted for, we still cannot exclude the possibility of residual or unmeasured confounding given the observational study design of the present analysis. Second, further studies are required to determine whether the findings from the present study including only Chinese patients can be extrapolated to other ethnic groups. Third, due to the shortage of records on insulin, we could not compare the TyG index with HOMA-IR and the hyperinsulinemic–euglycemic clamp test.

## Conclusions

In conclusion, our study shows that an elevated TyG index at baseline and in the long-term is independently associated with increased risk of MI, especially in women. Our findings indicate that this simple index may be useful for identifying individuals at high risk of developing MI at an early stage, and emphasize the importance of monitoring the TyG index in the long term in clinical practice.

## Supplementary Information

**Additional file 1: Table S1.** Baseline characteristics of excluded and included participants. **Table S2.** Proportionality assumptions test. **Table S3.** Baseline characteristics according to quartiles of updated mean TyG index. **Figure S1.** Flowchart of the study. **Figure S2.** Receiver operative characteristics curve and cutoff value of triglyceride-glucose index for incident myocardial infarction.

## Data Availability

The datasets used and/or analyzed during the current study are available from the corresponding author on reasonable request.

## Abbreviations

BMI: Body mass index
CI: Confidence interval
CVD: Cardiovascular disease
DBP: Diastolic blood pressure
FBG: Fasting blood glucose
HDL-C: High-density lipoprotein cholesterol
HOMA-IR: Hemeostasis model assessment of IR
HR: Hazard ratio
hs-CRP: High-sensitivity C-reactive protein
IQR: Interquartile range
IR: Insulin resistance
LDL-C: Low-density lipoprotein cholesterol
MI: Myocardial infarction
ROC: Receiver operative characteristic
SBP: Systolic blood pressure
TC: Total cholesterol
TG: Triglyceride
TyG: Triglyceride-glucose
