# Supplementary material for: Triglyceride–glucose index is associated with the risk of myocardial infarction: an 11-year prospective study in the Kailuan cohort
Source: Cardiovasc Diabetol. 2021 Jan 12;20:19. doi: 10.1186/s12933-020-01210-5 (PMC7802156; doi:10.1186/s12933-020-01210-5)
Supplement: Supplementary file 1 — Additional file 1: Table S1. Baseline characteristics of excluded and included participants. Table S2. Proportionality assumptions test. Table S3. Baseline characteristics according to quartiles of updated mean TyG index. Figure S1. Flowchart of the study. Figure S2. Receiver operative characteristics curve and cutoff value of triglyceride-glucose index for incident myocardial infarction. [file 12933_2020_1210_MOESM1_ESM.docx]

Table S1. Proportionality assumptions test for primary exposure variables

|  | Wald χ^2^ | *P* value |
| --- | --- | --- |
| Baseline TyG index | 5.41 | 0.1438 |
| Updated mean of TyG index | 18.76 | 0.0003 |
| Updated mean of TyG index*log(t) | 2.01 | 0.1565 |
| Number of visits with high TyG index | 0.98 | 0.8062 |

Abbreviation: TyG index, triglyceride-glucose index

Table S2. Baseline characteristics according to quartiles of the updated mean TyG index

| Characteristics | Quartiles of updated mean TyG index | | | | *P* value |
| --- | --- | --- | --- | --- | --- |
|  | Q1 | Q2 | Q3 | Q4 |  |
| No. of participants | 24712 | 22712 | 22713 | 22712 |  |
| TyG index | 8.00(7.74-8.23) | 8.43(8.22-8.63) | 8.77(8.53-9.02) | 9.34(9.02-9.76) | <0.0001 |
| Age, years | 51.70(42.7-60.49) | 51.91(43.66-59.41) | 52.13(44.15-59.16) | 51.36(43.69-57.84) | <0.0001 |
| Male, n (%) | 18742 (76.08) | 19569 (79.43) | 19840 (80.53) | 20416 (82.87) | <0.0001 |
| High school or above, n (%) | 5389 (22.58) | 4575 (19.18) | 4652 (19.58) | 4526 (19.06) | <0.0001 |
| Income >800 RMB/month, n (%) | 3505 (14.70) | 3268 (13.71) | 3395 (14.31) | 3447 (14.52) | 0.0139 |
| Body mass index, kg/m^2^ | 23.05(21.05-25.26) | 24.39(22.4-26.61) | 25.43(23.43-27.64) | 26.37(24.34-28.50) | <0.0001 |
| Systolic blood pressure, mm Hg | 120.70(110.00-138.70) | 129.30(116.70-140.00) | 130.00(120.00-145.00) | 130.70(120.00-150.00) | <0.0001 |
| Diastolic blood pressure, mm Hg | 80.00(70.70-86.00) | 80.00(76.70-90.00) | 81.00(79.30-90.00) | 84.00(80.00-91.70) | <0.0001 |
| Current smoker, n (%) | 8617 (35.88) | 8340 (34.72) | 9098 (37.97) | 9803 (40.80) | <0.0001 |
| Current alcohol use, n (%) | 21917 (92.03) | 21754 (91.38) | 21523 (90.85) | 21547 (90.88) | <0.0001 |
| Active physical activity, n (%) | 480 (1.95) | 540 (2.19) | 611 (2.48) | 671 (2.72) | <0.0001 |
| Diabetes Mellitus, n (%) | 148 (0.61) | 347 (1.41) | 650 (2.64) | 1855 (7.53) | <0.0001 |
| Hypertension, n (%) | 1834 (7.44) | 2592 (10.52) | 3429 (13.92) | 4349 (17.65) | <0.0001 |
| Dyslipidemia, n (%) | 667 (2.71) | 1092 (4.43) | 1527 (6.20) | 2432 (9.87) | <0.0001 |
| Antihypertensive drugs, n (%) | 100 (0.41) | 242 (0.98) | 511 (2.07) | 1428 (5.80) | <0.0001 |
| Antidiabetic drugs, n (%) | 78 (0.32) | 150 (0.61) | 217 (0.88) | 395 (1.60) | <0.0001 |
| Lipid-lowering drugs, n (%) | 1570 (6.37) | 2247 (9.12) | 3001 (12.18) | 3763 (15.27) | <0.0001 |
| Fasting plasma glucose, mmol/L | 4.84(4.45-5.26) | 5.02(4.60-5.50) | 5.20(4.72-5.80) | 5.60(5.00-6.70) | <0.0001 |
| Total cholesterol, mmol/L | 4.61(4.06-5.20) | 4.87(4.26-5.46) | 5.02(4.38-5.69) | 5.21(4.53-5.94) | <0.0001 |
| Triglycerides, mmol/L | 0.76(0.60-0.97) | 1.13(0.92-1.38) | 1.51(1.19-1.93) | 2.42(1.74-3.54) | <0.0001 |
| HDL cholesterol, mmol/L | 1.55(1.31-1.81) | 1.53(1.30-1.78) | 1.49(1.27-1.74) | 1.45(1.24-1.71) | <0.0001 |
| LDL cholesterol, mmol/L | 2.14(1.63-2.71) | 2.36(1.89-2.80) | 2.40(1.96-2.90) | 2.39(1.85-2.90) | <0.0001 |
| Hs-CRP, mg/dL | 0.60(0.20-1.70) | 0.73(0.29-2.00) | 0.90(0.34-2.30) | 1.10(0.46-2.75) | <0.0001 |

Abbreviations: LDL, low-density lipoprotein; HDL, high-density lipoprotein; hs-CRP, high-sensitivity C-reactive protein; TyG, triglyceride glucose

Table S3. Baseline characteristics of excluded and included participants

| Characteristics | Excluded | Included | ASD/HL estimator, % | *P* value |
| --- | --- | --- | --- | --- |
| No. of participants | 43723 | 55452 |  |  |
| Age, years | 54.56(48.02-62.78) | 49.58(41.86-56.58) | 44.61 | <0.0001 |
| Male, n (%) | 36757 (84.07) | 42311 (76.30) | 19.58 | <0.0001 |
| High school or above, n (%) | 6520 (15.62) | 12663 (23.66) | 20.36 | <0.0001 |
| Income >800 RMB/month, n (%) | 5252 (12.59) | 8366 (15.65) | 8.80 | <0.0001 |
| Body mass index, kg/m2 | 24.79(22.58-27.13) | 24.90(22.65-27.25) | 2.89 | <0.0001 |
| Systolic blood pressure, mm Hg | 130.00(120.00-149.30) | 125.30(115.00-140.00) | 25.60 | <0.0001 |
| Diastolic blood pressure, mm Hg | 80.70(79.30-90.00) | 80.00(76.70-90.00) | 16.30 | <0.0001 |
| Current smoker, n (%) | 14332 (34.05) | 18617 (34.52) | 1.00 | 0.1233 |
| Current alcohol use, n (%) | 14415 (34.23) | 21442 (39.75) | 11.45 | <0.0001 |
| Active physical activity, n (%) | 38074 (91.35) | 48692 (91.20) | 0.51 | 0.4356 |
| Diabetes Mellitus, n (%) | 1510 (3.45) | 1488 (2.68) | 4.47 | <0.0001 |
| Hypertension, n (%) | 5989 (13.70) | 6120 (11.04) | 8.09 | <0.0001 |
| Dyslipidemia, n (%) | 2366 (5.41) | 3300 (5.95) | 2.33 | <0.0001 |
| Antihypertensive drugs, n (%) | 1130 (2.58) | 1153 (2.08) | 7.19 | <0.0001 |
| Antidiabetic drugs, n (%) | 334 (0.76) | 496 (0.89) | 3.35 | <0.0001 |
| Lipid-lowering drugs, n (%) | 5167 (11.82) | 5321 (9.60) | 1.44 | 0.0250 |
| Total cholesterol, mmol/L | 4.96(4.30-5.61) | 4.90(4.26-5.57) | 3.71 | <0.0001 |
| HDL cholesterol, mmol/L | 1.50(1.27-1.76) | 1.51(1.29-1.77) | 1.18 | <0.0001 |
| LDL cholesterol, mmol/L | 2.40(1.88-2.90) | 2.30(1.77-2.79) | 13.48 | <0.0001 |
| Hs-CRP, mg/dL | 0.88(0.32-2.20) | 0.78(0.30-2.20) | 1.91 | <0.0001 |

Abbreviations: ASD, absolute standardized difference; LDL, low-density lipoprotein; HDL, high-density lipoprotein; hs-CRP, high-sensitivity C-reactive protein; TyG, triglyceride glucose; HL estimator: Hodges-Lehmann estimator

Continuous variables were presented as means along with standard deviations or medians along with inter-quartile range. Categorical variables were presented as percentages. Given the large study population in our study, *P*<0.05 in the comparison indicates statistical significance but may not mean clinical significance. Therefore, baseline characteristics between excluded and included participants were compared with ASD for means or percentages, and HL estimator for medians, the indicator >10% is approximately equivalent to *P* value less than 0.05, indicating a significant imbalance

Figure S1. Flowchart of the study

Abbreviations: FBG, fasting blood glucose; MI, myocardial infarction; TG, triglyceride; TyG, triglyceride-glucose


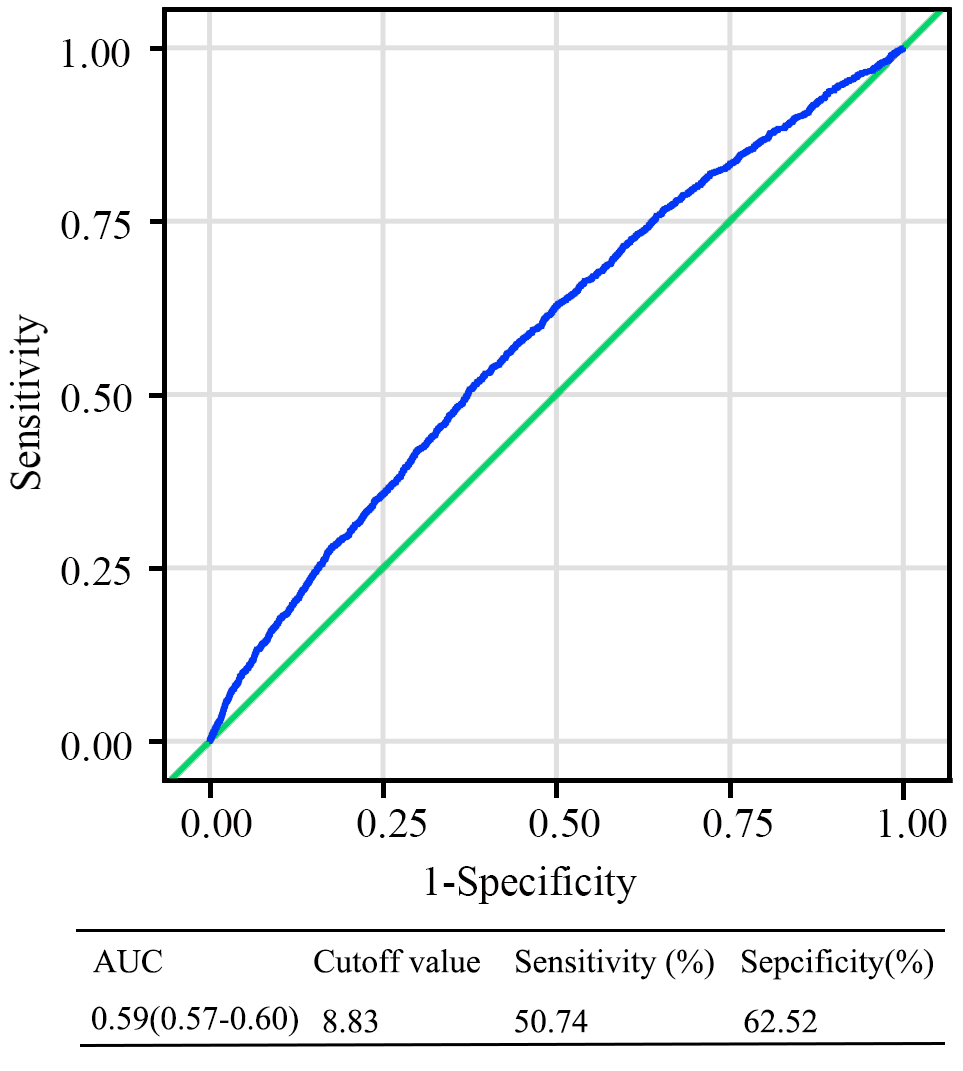


Figure S2. Receiver operative characteristics curve and cutoff value of triglyceride-glucose index for incident myocardial infarction.

Abbreviation: AUC, area under curve
